# Ag@AuNP-Functionalized Capillary-Based SERS Sensing Platform for Interference-Free Detection of Glucose in Urine Using SERS Tags with Built-In Nitrile Signal

**DOI:** 10.3390/molecules28247939

**Published:** 2023-12-05

**Authors:** Yanmei Si, Hua Wang, Yehao Yan, Bingwen Li, Zeyun Ni, Hongrui Shi

**Affiliations:** 1 Institute of Forensic Medicine and Laboratory Medicine, Jining Medical University, Jining 272067, China; 2 School of Life Science, Huzhou University, Huzhou 313000, China; 3 School of Public Health, Jining Medical University, Jining 272067, China; 4 Shandong Key Laboratory of Biophysics, Institute of Biophysics, Dezhou University, Dezhou 253023, China

**Keywords:** SERS sensing analysis, functionalized capillary, Ag@AuNPs, SERS tags, interference-free signal, glucose

## Abstract

A Ag@AuNP-functionalized capillary-based surface-enhanced Raman scattering (SERS) sensing platform for the interference-free detection of glucose using SERS tags with a built-in nitrile signal has been proposed in this work. Capillary-based SERS capture substrates were prepared by connecting 4-mercaptophenylboronic acid (MBA) to the surface of the Ag@AuNP layer anchored on the inner wall of the capillaries. The SERS tags with a built-in interference-free signal could then be fixed onto the Ag@AuNP layer of the capillary-based capture substrate based on the distinguished feature of glucose, which can form a bidentate glucose–boronic complex. Thus, many “hot spots” were formed, which produced an improved SERS signal. The quantitative analysis of glucose levels was realized using the interference-free SERS intensity of nitrile at 2222 cm^−1^, with a detection limit of about 0.059 mM. Additionally, the capillary-based disposable SERS sensing platform was successfully employed to detect glucose in artificial urine, and the new strategy has great potential to be further applied in the diagnosis and control of diabetes.

## 1. Introduction

Diabetes as a lethal diseases is caused by a deficiency of insulin production in the pancreas, which induces abnormal high glucose levels in the body [1,2,3,4]. Glucose is an indispensable nutrient for human metabolism. High glucose levels are closely related to the progression of diabetes, while low glucose levels can cause hypoglycemia or insulin shock [5,6,7]. To effectively control the disease, the glucose level in the blood needs to be tested frequently, which causes an inconvenience to patients. Urine samples are not a direct substitute for blood sample in monitoring glucose levels for diabetes but are rather an alternative or complement that can provide valuable information when blood is not accessible or using it is not desirable [8].

Among the existing detection methods, electrochemical and fluorescence detection methods are the most successful methods for the clinical detection of glucose levels [9,10,11,12,13]. However, most of these methods require the use of biological enzymes with poor storage stability and low biological activity, which affects the accuracy of analysis [14,15]. Thus, it is an urgent need to develop a flexible detection method for the interference-free sensing of glucose. The surface-enhanced Raman scattering (SERS) detection technique can exhibit sensitivity at the single-molecule level, and has been widely used in biology, chemistry, medicine, and other fields [16,17,18]. Van Duyne’s group presented the direct SERS detection of glucose based on the partition of glucose into an alkanethiol monolayer adsorbed on silver film [19,20]. Although these methods achieved good results, they were limited by the small Raman interface of glucose molecules, and the non-specific capture of glucose. The Raman signals of molecules such as alkyne, nitrile, and cyanide ion in the silent region (1800–2800 cm^−1^) did not overlap with background signals, avoiding interference from those of regular molecules [21,22,23,24]. Thanks to the development of nanomaterials, many new SERS substrates were prepared for SERS sensing detection, and satisfactory detection results were obtained [25,26,27]. For example, Bandarenka’s group prepared gold-coated porous silicon nanostructures for molecular analysis via SERS spectroscopy [26]. Notably, molecules with Raman signals in the silent region could be embedded into nanomaterials with core–shell structures to prepare SERS tags, which greatly expanded their application in SERS detection technology [28,29,30]. Raman marker molecules were anchored in the core–shell interface of the nanomaterial and were not exposed to the external environment, avoiding displacement by other molecules. In addition, a completely free shell surface was available to come in contact with the other molecule [31,32,33]. Moreover, this kind of SERS tag with the built-in SERS signal did not affect the spatial distribution of target molecule capture and recognition.

As is well-known, the recognition element is an important component of biosensors for the detection of biologically related substances. Boric acid, as a versatile molecule, can form five-membered ring complexes with 1, 2-diols or six-membered ring complexes with 1, 3-diols by forming reversible covalent bonds [34]. Based on this unique borate–diol interaction, many colorimetric, fluorescent, and electrochemical sensors have been developed for the determination of diol-containing substances [35,36,37,38,39,40]. Liu’s group established a magnetic bead-based electrochemical and colorimetric strategy for sensing circulating tumor cells using boronic acid derivatives as the recognition elements and signal probes [36]. In addition, this borate–diol interaction has been used in non-enzymatic glucose sensing [40]. However, due to the affinity of boric acid to other monosaccharides such as fructose and galactose, the selectivity of these detection methods for glucose are limited to some extent. Considering that glucose can form a bidentate glucose–boronic complex, a series of selective detection methods for glucose have been constructed [41]. For example, Bi’s group established a SERS-based glucose sandwich assay using 4,4′-dimercaptoazobenzene as the actual Raman reporter [42]. This method improved the selectivity of glucose detection, but the signal molecules were exposed and easily replaceable, and the Raman signal was located in the fingerprint region, which was susceptible to environmental interference. Based on this, if SERS tags with a built-in non-interference signal can be combined with the unique property of glucose, which is that it can form a bidentate glucose–boronic complex, the selectivity and anti-interference performance of glucose sensing will be greatly improved. In addition, the capillary-based SERS sensing platform has the characteristics of low cost, the possibility of miniaturization, easy portability, and the ability to extract microsamples, making it an ideal platform for portable microanalysis [43,44].

**Scheme 1:**
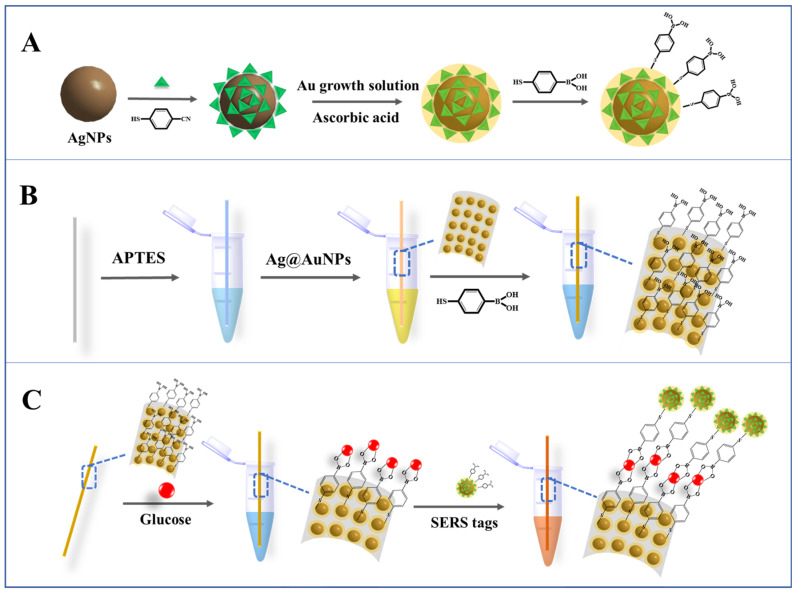
Schematic illustration of (**A**) the preparation of SERS tags, (**B**) the fabrication of capillary-based SERS capture substrate, (**C**) the capillary-based SERS sensing platform for the detection of glucose by using SERS tags with a built-in interference-free signal.

Herein, we propose a disposable Ag@AuNP-functionalized capillary-based SERS sensing platform for the detection of glucose using SERS tags with a built-in interference-free signal (Scheme 1). Firstly, based on the synthesis of Ag@AuNPs, SERS tags (Ag@MBN@AuNPs) with built-in Raman signals in the silent region and SERS-enhanced properties were prepared by introducing the marker molecule 4-mercaptobenzonitrile (MBN). The Ag@AuNPs were modified onto the inner wall of the capillary, and 4-mercaptophenylboronic acid (MBA) was further modified on the surface of the Ag@AuNP layer to prepare a disposable SERS capture substrate for the recognition of glucose. MBA was modified on the surface of Ag@MBN@AuNPs to form SERS tags as the signal probe with SERS-enhanced performance. In the presence of glucose, a bidentate glucose–boronic complex was formed [45,46,47], connecting the two boronic acid groups of the capillary-based capture substrate and the SERS tag signal probe. Thus, the SERS tags with an undisturbed SERS signal (2222 cm^−1^) were anchored onto the capillary-based capture substrate. In addition, “hot spots” were generated due to the interactions between the SERS tags and the Ag@AuNP layer on the inner wall of the capillary, which ultimately produced a strong SERS signal that further improved the detection sensitivity toward glucose.

## 2. Results and Discussion

### 2.1. Characterization of the Ag@AuNPs and Ag@MBN@AuNPs

**Figure 1:**
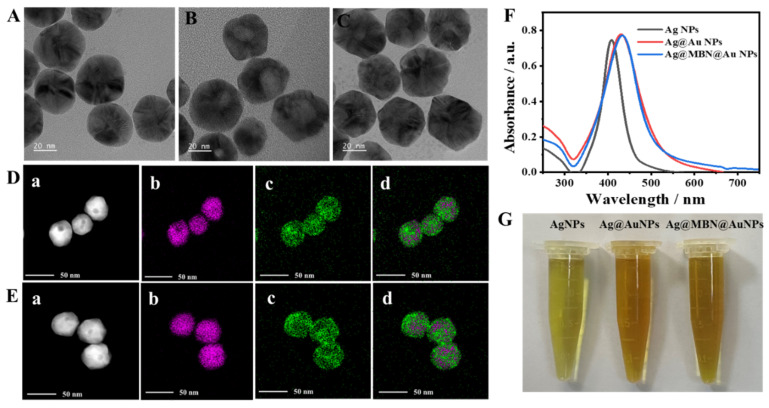
TEM images of (**A**) AgNPs. (**B**) Ag@AuNPs, and (**C**) Ag@MBN@AuNPs. (**D**) Elemental analysis of Ag@AuNPs: (**a**) DF-STEM image, (**b**,**c**) EDX elemental maps of Ag and Au, and (**d**) overlay of Ag and Au. (**E**) Elemental analysis of Ag@MBN@AuNPs: (**a**) DF-STEM image, (**b**,**c**) EDX elemental maps of Ag and Au, and (**d**) overlay of Ag and Au. Notes: red color represent the Ag element and green color represent the Au element in Figure (**D**,**E**). (**F**) UV−vis spectra of AgNPs, Ag@AuNPs, and Ag@MBN@Au NPs. (**G**) Photographs of AgNP, Ag@AuNP, and Ag@MBN@AuNP solutions.

TEM images of the AgNPs, Ag@AuNPs, and Ag@MBN@AuNPs are shown in Figure 1A–C, respectively. As we can see, the spherical structure of AgNPs with uniform diameter distribution were obtained. The TEM images present the morphology of the AgNPs with an average diameter of about 44 nm (Figure S1; details are in the Supporting Information (SI)). In comparison, the morphology of the Ag@AuNPs and Ag@MBN@AuNPs did not change significantly, while their average diameter increased slightly to about 46 nm (Figures S2 and S3). Additionally, the results of elemental analysis showed that there were obvious gold elements on the surfaces of the AgNPs, forming the core–shell structure of the Ag@AuNPs (Figure 1D) and Ag@MBN@AuNPs (Figure 1E). Moreover, the presence of Au in the energy spectrum analysis (Figure S4) and energy spectrum quantification report (Table S1) further verified the successful preparation of Ag@AuNPs and Ag@MBN@AuNPs.

In addition, the three kinds of nanoparticles were compared using the UV–vis spectra in Figure 1F. The absorption peaks of Ag@AuNPs (with maximum absorption wavelengths at 430 nm) and Ag@MBN@AuNPs (with maximum absorption wavelengths at 434 nm) showed an obvious red shift compared with the absorption peak of the AgNPs (with maximum absorption wavelengths at 408 nm). The results are consistent with those reported in the literature that say that the growth of the Au shell on the surface of AgNPs could induce a red shift in the absorption peak [48]. The photographs in Figure 1G show that the color of the Ag@AuNP solution was similar to that of the Ag@MBN@AuNP solution, and the colors of both were darker than that of the AgNP solution. All these phenomena indicated the successful preparation of the core–shell structure Ag@AuNPs and Ag@MBN@AuNPs. As is well known, stability is an essential consideration in the selection of an ideal SERS substrate. Therefore, the stability of three kinds of nanoparticles was tested in H_2_O_2_ solution (Figure S5). The absorption peak of AgNPs disappeared after 2 h of etching. On the contrary, the UV−vis spectra of the core–shell structure Ag@AuNPs and Ag@MBN@AuNPs remained at their original intensity after the same treatment. The comparative results suggest that the Au shell greatly improved the stability of the AgNPs, which may expand their potential use in many plasmon-based applications, especially when corrosive reagents are involved.

### 2.2. Establishment of Ag@AuNP-Functionalized Capillary-Based SERS Capture Substrate

**Figure 2:**
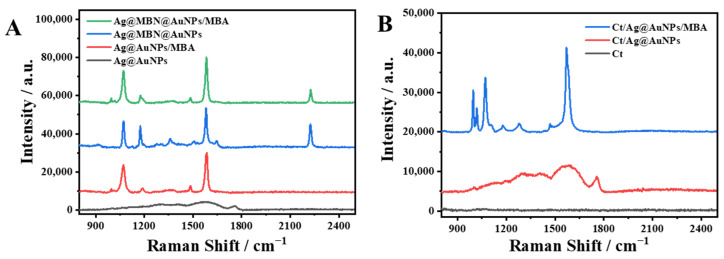
(**A**) SERS spectra of Ag@AuNPs, Ag@AuNPs/MBA, Ag@MBN@AuNPs, and Ag@MBN@AuNPs/MBA. (**B**) SERS spectra of capillary (Ct), capillary/Ag@AuNPs (Ct/Ag@AuNPs), and capillary/Ag@AuNPs/MBA (Ct/Ag@AuNPs/MBA).

Before the construction of the capillary-based SERS sensing platform, the SERS properties of Ag@AuNPs and Ag@MBN@AuNPs were first investigated (Figure 2A). SERS signals at 993, 1070, and 1582 cm^−1^ derived from the characteristic vibrational modes of MBA were observed for Ag@AuNPs after modification with MBA (the SERS band assignment was summarized and is shown in Table S2), indicating their successful immobilization on the surface of Ag@AuNPs. For Ag@MBN@AuNPs, a strong SERS signal derived from the nitrile group of MBN was observed at 2222 cm^−1^, suggesting that it can be used as a SERS tag with a Raman signal that is not disturbed by a conventional interfering component. Further examination revealed that Raman scattering peaks at 993 cm^−1^ and at 2222 cm^−1^ could be observed simultaneously after the self-assembly of MBA on the surface of the Ag@MBN@AuNPs. The results demonstrated the successful preparation of Ag@MBN@AuNPs/MBA with an interference-free nitrile signal and the ability for glucose recognition. In addition, in order to ensure the stability of Ag@AuNPs and Ag@MBN@AuNPs during the MBA-modified process, UV–vis spectra before and after MBA modification were recorded. As shown in Figure S6, the UV–vis spectra of both Ag@AuNPs and Ag@MBN@AuNPs did not noticeably change after MBA modification, suggesting that the agglomeration of nanoparticles did not occur. The results further indicated that the process of MBA connecting to the particle surface did not affect the stability of the nanoparticles.

After ensuring the SERS properties of the nanomaterials, the capillaries were gradually treated and modified. The aminosilylated inner wall of the capillaries was further incubated with the Ag@AuNPs. One can see from the photograph of the capillary that a significant earthy yellow color can be observed for the Ag@AuNP-treated aminosilylated capillary (Figure S7a). In contrast, no color change was found in the raw capillary (untreated by APTES) after incubation with Ag@AuNPs (Figure S7b). Further SERS comparison (Figure 2B) showed that there was no obvious SERS signal in the capillary alone (Ct) or in the capillary after the modification of Ag@AuNPs (Ct/Ag@AuNPs). Only after further modification with MBA (Ct/Ag@AuNPs/MBA) was an obvious characteristic SERS signal derived from the MBA was observed. These comparative results all indicated the successful construction of the capillary-based SERS capture substrate.

**Figure 3:**
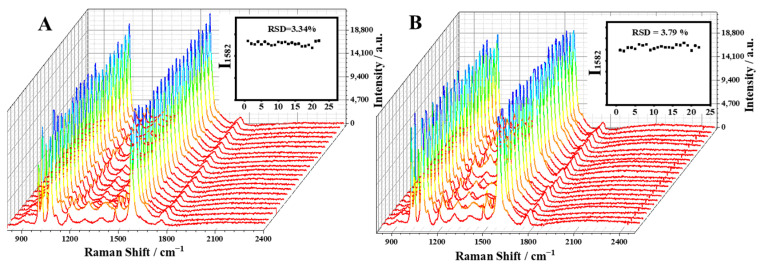
(**A**) SERS spectra from 22 different points of one capillary-based SERS capture substrate. (Insert: standard deviation of SERS intensity at 1582 cm^−1^ for the 22 SERS spectra in (**A**)). (**B**) SERS spectra from 22 different capillary-based SERS capture substrates. (Insert: standard deviation of SERS intensity at 1582 cm^−1^ for the 22 SERS spectra in (**B**)).

The signal homogeneity and reproducibility of the capillary-based SERS capture substrate were crucial factors affecting the practical application of the SERS sensing analysis. The SERS spectra of 22 random points selected from the same capillary-based SERS capture substrate were recorded. Figure 3A showed that the SERS intensities at 1582 cm^−1^ of 22 SERS spectra were basically the same. According to the calculations, the relative standard deviation (RSD) of the SERS signal intensity at 1582 cm^−1^ was 3.34%. Then, the SERS intensities at 1582 cm^−1^ from 22 different parallelly prepared capillary-based SERS capture substrates were compared. Figure 3B showed that the SERS signal intensity at 1582 cm^−1^ was similar for the 22 parallelly capturing substrates and the RSD was 3.79%. Therefore, the capillary-based SERS capture substrate had satisfactory uniformity and reproducibility, which ensured the reliability of the SERS sensing analysis during the glucose assay in practical applications.

### 2.3. Feasibility of Glucose Analysis by the Capillary-Based SERS Sensing Platform and Optimization of Main Detection Conditions

**Figure 4:**
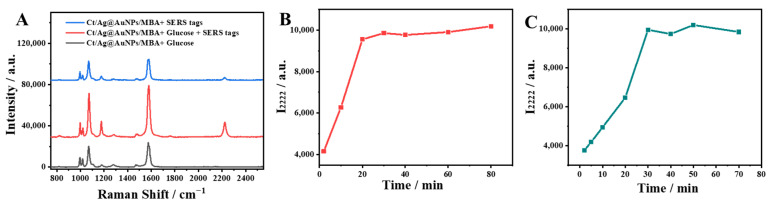
(**A**) SERS spectra of Ct/Ag@AuNPs/MBA + Glucose, Ct/Ag@AuNPs/MBA + Glucose + SERS tags, and Ct/Ag@AuNPs/MBA + SERS tags. Effects of incubation time for (**B**) glucose and (**C**) SERS tags.

The feasibility of the capillary-based SERS capture platform for the determination of glucose levels was also evaluated (Figure 4A). There was no significant SERS signal in the range of 1800–2500 cm^−1^ when SERS tags were incubated with the capillary-based SERS capture substrate in the absence of glucose. In contrast, a distinct Raman scattering peak at 2222 cm^−1^, attributed to the nitrile group of the SERS tags, was observed on the capillary-based SERS sensing platform in the presence of glucose and SERS tags. These contrasting results demonstrated the unique feature of glucose, which is that it can form a bidentate glucose–boronic complex between SERS tags and the capillary-based SERS capture substrate. “Hot spots” are regions where surface plasmons are highly localized in a small volume of the order of a few nm, giving rise to very large SERS enhancements [49,50]. Theoretical simulations were conducted and verified the formation of “hot spots” when SERS tags were bound to the Ag@AuNP layer through the bridging role of glucose (Figure S8). These “hot spots” further enhanced the SERS signals, suggesting the feasibility of the capillary-based SERS sensing platform for the sensitive analysis of glucose.

The experimental conditions were optimized to produce a stronger SERS signal for optimal glucose detection performance. The effect of the contacting time (2–80 min) of glucose solution with the capillary-based SERS capture substrate was investigated (Figure 4B). The SERS intensity at 2222 cm^−1^ increased sharply with the increasing time before 20 min and then remained constant. Therefore, 20 min was selected as the optimal reaction time. Similarly, the reaction time of the SERS tags was also considered (2–70 min), and 30 min was used for subsequent detection (Figure 4C).

### 2.4. SERS Determination of Glucose Levels Using the Capillary-Based SERS Sensing Platform

**Figure 5:**
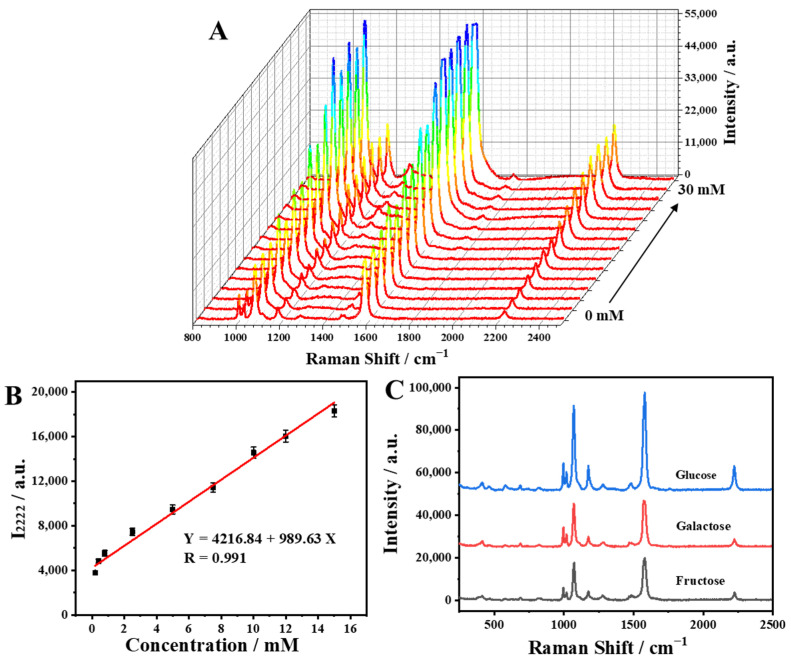
(**A**) SERS spectra of the capillary-based SERS sensing platform’s response to different concentrations of glucose. (**B**) Linear relationship between the SERS intensity (I_2222_) of the capillary-based SERS sensing platform and glucose concentration. (**C**) SERS spectra of the capillary-based SERS sensing platform incubated with glucose, galactose, and fructose at the same concentrations (5 mM).

The capability of the capillary-based SERS sensing platform for the analysis of glucose was evaluated in buffer solution (Figure 5A). The SERS intensity at 2222 cm^−1^, attributed to the nitrile group of the SERS tags, increased with an increasing concentration of glucose. Further investigation revealed that there was a good linear relationship between the peak intensity at 2222 cm^−1^ and the concentration of glucose over the range 0.2–15 mM (Figure 4B). Moreover, a detection limit of about 0.059 mM was estimated in accordance with the 3σ rule. It is worth noting that the detection range met the requirements for the clinical detection of glucose [42,51]. To examine the selectivity of the sensing platform for glucose, its responses to other saccharides including fructose and galactose were also tested. As can be seen in Figure 5C, only glucose could trigger a pronounced signal change at 2222 cm^−1^. The weak SERS signal of fructose and galactose may have been due to their lower tendency to form bidentate complexes and the small amount of non-specific adsorption of SERS tags [45,46,47]. Although fructose and galactose have greater affinities for monoboronic acids to form 1:1 complexes (the binding affinity of fructose to boronic acid is 1~2 orders higher than that of glucose), glucose has the ability of binding to double boronic acids to form 1:2 bidentate complexes [41,42,51,52]. Therefore, the procedure avoided signal interference from other sugars and improved the selectivity of the capillary-based SERS sensing platform. Moreover, the concentration of glucose (~5 mM) in human blood is almost two orders of magnitude higher than that of fructose (~0.05 mM) and galactose (~0.05 mM) [42,52], which further ensured that the capillary-based SERS sensing platform for glucose detection was not interfered with by fructose and galactose.

### 2.5. Practical Analysis of Glucose in Artificial Urine

To further verify the feasibility of the proposed capillary-based SERS sensing strategy for the detection of glucose in real samples, different amounts of glucose were added into artificial urine samples, and the concentration of glucose in each sample was determined. The recovery rate was calculated and is summarized in Table 1, and it was in the range of 98.17–108.55%, suggesting that the capillary-based SERS sensing platform proposed in this work exhibits certain reliability for the determination of glucose in complex systems.

## 3. Experimental Section

### 3.1. Materials and Methods

#### 3.1.1. Chemical Reagents

Auricchloridedihydrate (HAuCl_4_) and silver nitrate (AgNO_3_) were purchased from Sinopharm Chemical Reagent Co., Ltd. (Shanghai, China). Sodium borohydride (NaBH_4_), L-ascorbic acid, and poly(vinylpyrrolidone) (PVP, Mw = 10,000) were obtained from Sigma−Aldrich (Shanghai, China) Trading Co., Ltd. (Shanghai, China). Sodium sulfite (Na_2_SO_3_), mercaptophenylboric acid (MBA), mercaptobenzonitrile (MBN), (3-aminopropyl) triethoxysilane (APTES), ethanol, acetone, acetonitrile (CH_3_CN), glucose, fructose, galactose, urea, and uric acid were supplied by J&K Scientific (Beijing, China). The ultrapure water (>18 MΩ) used in all assays was obtained from an ultrapure water system (Wuhan Youpu Instrument Equipment Co., Ltd., Wuhan, China).

#### 3.1.2. Characterizations

Transmission electron microscopy (TEM) images were acquired with a transmission electron microscope (JEOL, JEM-F200 (HR)). Absorption spectra were measured using a Hitachi UH-5700 spectrophotometer (Kyoto, Japan). SERS spectra were recorded with a portable Raman spectrometer (BWS415-532S, B&W Tek, Inc., Newark, DE, USA). 

#### 3.1.3. SERS Detection Parameters

The SERS spectra were acquired using a 532 nm excitation laser with an acquisition time of 3 s. All of the spectra in this work were baseline-corrected.

### 3.2. Preparation of Ag@AuNPs

#### 3.2.1. AgNPs

AgNPs were synthesized through the seed growth method [53]. Initially, a 5.0 mL volume of PVP solution (w% = 5.0%) was mixed with a 5.0 mL volume of HAuCl_4_ solution (0.50 mM), and then a 0.6 mL volume of NaBH_4_ solution (0.1 M) was added into the mixture. The solution was then used as a seed solution after standing and aging for 6 h. The Ag growth solution was prepared by adding 10 mL of PVP solution (5%), 5 mL of CH_3_CN solution, 1 mL of L-ascorbic acid (0.1 M) solution, and 0.75 mL of AgNO_3_ solution (0.1 M) into 10 mL of ultrapure water. Under intense agitation, 20 µL of the prepared seed solution was quickly added to the Ag growth solution. After 30 min, AgNPs were collected via centrifugation (10,000 rpm, 8 min) and re-dispersed with 10 mL of ultrapure water.

#### 3.2.2. Ag@AuNPs

Ag@AuNPs were synthesized through a modified synthetic procedure reported previously [53]. Under intense agitation, 4.72 mL of ultrapure water, 40 µL of HAuCl_4_ solution (0.25 M), 240 µL of NaOH solution (0.2 M), and 3.0 mL of Na_2_SO_3_ solution (0.01 M) were added to a 50 mL centrifuge tube. After 10 min of continuous stirring, the solution was placed at 4 °C overnight and used as Au growth solution. An amount of 1 mL of AgNP solution was centrifuged (at 10,000 rpm for 5 min) and dispersed with 1 mL of PVP solution (w% = 5.0%). Then, 7 mL of PVP solution (w% = 5.0%) was added into the AgNP solution and stirred to mix well. After that, 4 mL of Au growth solution, 200 µL of L-ascorbic acid solution (0.5 M), 200 µL of NaOH solution (0.5 M), and 200 µL of Na_2_SO_3_ (0.1 M) were added successively, and the reaction was maintained at 37 °C for 6 h. After that, Ag@AuNPs were collected via centrifugation (at 10,000 rpm for 8 min), and re-dispersed with 2 mL of PVP solution (w% = 5.0%).

### 3.3. Preparation of SERS Tags (Ag@MBN@AuNPs@MBA)

AgNPs were modified with MBN (0.1 mM) for 12 h. The MBN-modified AgNPs were collected via centrifugation (10,000 rpm, 8 min), and then re-dispersed into PVP solution (w% = 5.0%). The synthetic route for Ag@MBN@AuNPs was similar to that for Ag@AuNPs. After centrifugation (at 12,000 rpm for 4 min), 1 mL of Ag@MBN@AuNPs was re-dispersed into 0.9 mL of ultrapure water, and then 0.1 mL of MBA (1 mM) was added and incubated for 3 h. Finally, the SERS tags were obtained via centrifugation (at 12,000 rpm for 4 min) and re-dispersed into 1.0 mL of ultrapure water.

### 3.4. Fabrication of Capillary-Based SERS Capture Substrate

The functionalization of capillaries was carried out through a modified procedure reported previously [43,44]. The capillaries were respectively immersed in acetone and ethanol solution for 15 min, immersed in APTES solution (w% = 1.0%) overnight, and then washed with ethanol. The treated capillaries were dried in a vacuum drying oven at 40 °C. After that, the Ag@AuNP solution was absorbed by an amino silanized capillary, and after a reaction for 8 h, the solution was cleaned with ultrapure water three times to obtain the capillary/Ag@AuNPs. The capillary/Ag@Au@MBA NPs were prepared by further absorbing MBA solution (0.1 mM) with capillary/Ag@AuNPs, leaving it to react for 3 h, and washing it with ultrapure water three times. The capillary/Ag@Au@MBA NPs were used as the capillary-based SERS capture substrate for glucose detection in subsequent experiments.

### 3.5. SERS Analysis of Glucose Using the Capillary-Based SERS Sensing Platform

Capillary-based SERS capture substrates were used to absorb different concentrations of glucose in buffer solution, incubated for 20 min, and washed with ultrapure water three times. After that, the capillaries were treated to absorb SERS tags and incubated for 30 min, then washed with ultrapure water three times. Finally, SERS spectra of the capillary-based SERS sensing platform were recorded with a portable Raman spectrometer.

The artificial urine (mainly containing about 97% water, 1.8% urea, 0.004% uric acid, and 1.1% NaCl) was first prepared. Then, different concentrations of glucose were spiked into the artificial urine and used as a glucose solution. The detection of glucose in artificial urine was then performed as described above.

## 4. Conclusions

In summary, we successfully developed a disposable Ag@AuNP-functionalized capillary-based SERS sensing platform for the detection of glucose levels using SERS tags with a built-in interference-free signal. Ag@AuNPs with a thin gold shell structure ensured the satisfactory SERS-enhanced properties of AgNPs, improved their chemical stability, and provided the possibility of built-in Raman molecules. SERS tags with the built-in nitrile group signal protected signal molecules from being replaced and avoided signal interference from other components. SERS hot spots were formed between the Ag@AuNP layer and SERS tags based on the feature of glucose, which can form a bidentate glucose–boronic complex, enhancing SERS intensity and improving detection sensitivity. The use of disposable capillaries enabled the high flexibility and simplicity of the glucose sensing strategy. This strategy for the glucose assay combined the portability of the capillary, interference-free nature of SERS tags, the bridging role of target glucose, and the high sensitivity of hot spot-based SERS detection, showing great potential in monitoring glucose levels.

## Figures and Tables

**Table 1:** Determination of glucose levels in artificial urine using the capillary-based SERS sensing platform.

Sample	Analyte	Added (mM)	Found (mM)	Recovery (%)
Artificial urine	Glucose	1	1.0855 ± 0.0421	108.55
		2	2.0560 ± 0.2489	102.80
		5	4.9086 ± 0.7176	98.17
		10	10.7915 ± 1.4868	107.92

## Data Availability

The data that support the fundings of this study are available from the corresponding author upon reasonable request.

## Supplementary Materials

The following supporting information can be downloaded at https://www.mdpi.com/article/10.3390/molecules28247939/s1. Figure S1. TEM and size distribution of AgNPs; Figure S2. TEM and size distribution of Ag@AuNPs; Figure S3. TEM and size distribution of Ag@MBN@AuNPs; Figure S4. Energy spectrum of (A) Ag@AuNPs and (B) Ag@MBN@AuNPs; Table S1. Energy spectrum quantification report; Figure S5. UV−vis spectra of (A) AgNPs, (B) Ag@AuNPs and (C) Ag@MBN@AuNPs before and after treatment with H_2_O_2_. Table S2. SERS bands assignment of SERS spectra observed in Figure 2 and Figure 3; Figure S6. UV−vis spectra of (A) Ag@AuNPs and (B) Ag@MBN@AuNPs before and after modified by MBA; Figure S7. Photographs of aminosilylated capillary treated with Ag@AuNPs (a) and raw capillary treated with Ag@AuNPs (b); Figure S8. Simulated near-field electromagnetic field distributions of the SERS sensing platform. Refs. [29,54,55,56] are cited in SM file.
