# Supplementary material for: Ag@AuNP-Functionalized Capillary-Based SERS Sensing Platform for Interference-Free Detection of Glucose in Urine Using SERS Tags with Built-In Nitrile Signal
Source: Molecules. 2023 Dec 5;28(24):7939. doi: 10.3390/molecules28247939 (PMC10745321; doi:10.3390/molecules28247939)
Supplement: Supplementary file 1 [file molecules-28-07939-s001.zip › molecules-2725441-supplementary.pdf]

Supplementary Information for

Ag@AuNPs functionalized capillary-based SERS sensing platform for interference-free detection of glucose in urine by using SERS tags with built-in nitrile signal

Yanmei Si [1], Hua Wang [2,*], Yehao Yan [3,*] Bingwen Li [4], Zeyun Ni [4], Hongrui Shi [4]

[1] Institute of Forensic Medicine and Laboratory Medicine, Jining Medical University, Jining 272067, PR China.

[2] School of Life Science, Huzhou University, Huzhou, 313000, PR China.

[3] School of Public Health, Jining Medical University, Jining, Shandong, 272067, PR China.

[4] Shandong Key Laboratory of Biophysics, Institute of Biophysics, Dezhou University, Dezhou, 253023, P. R. China.

Table of Content

Figure S1. TEM and size distribution of AgNPs.

Figure S2. TEM and size distribution of Ag@AuNPs.

Figure S3. TEM and size distribution of Ag@MBN@AuNPs.

Figure S4 Energy spectrum of (A) Ag@AuNPs and (B) Ag@MBN@AuNPs.

Table S1 Energy spectrum quantification report.

Figure S5. UV−vis spectra of (A) AgNPs, (B) Ag@AuNPs and (C) Ag@MBN@AuNPs before and after treatment with H_2O_2.

Table S2 SERS bands assignment of SERS spectra observed in Figure. 2 and Figure. 3.

Figure S6. (A) UV−vis spectra of (A) Ag@AuNPs and (B) Ag@MBN@AuNPs before and after modified by MBA.

Figure S7. Photographs of aminosilylated capillary treated with Ag@AuNPs (a) and raw capillary treated with Ag@AuNPs (b).

Figure S8 Simulated near-field electromagnetic field distributions of the SERS sensing platform.

Additional figures

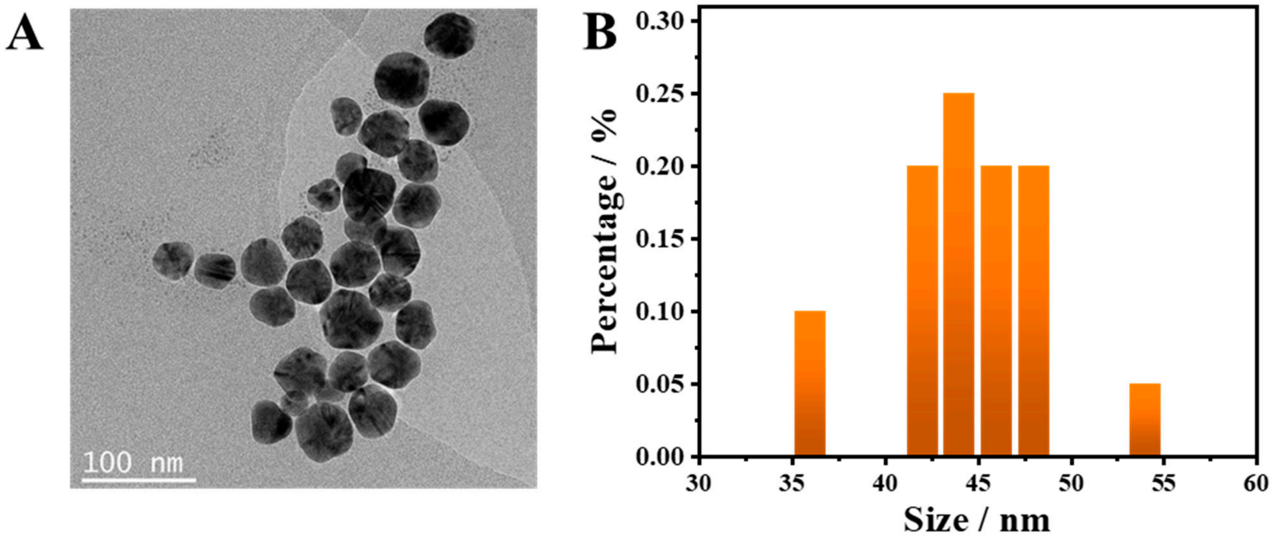

Figure S1 (A) TEM and (B) size distribution of AgNPs.

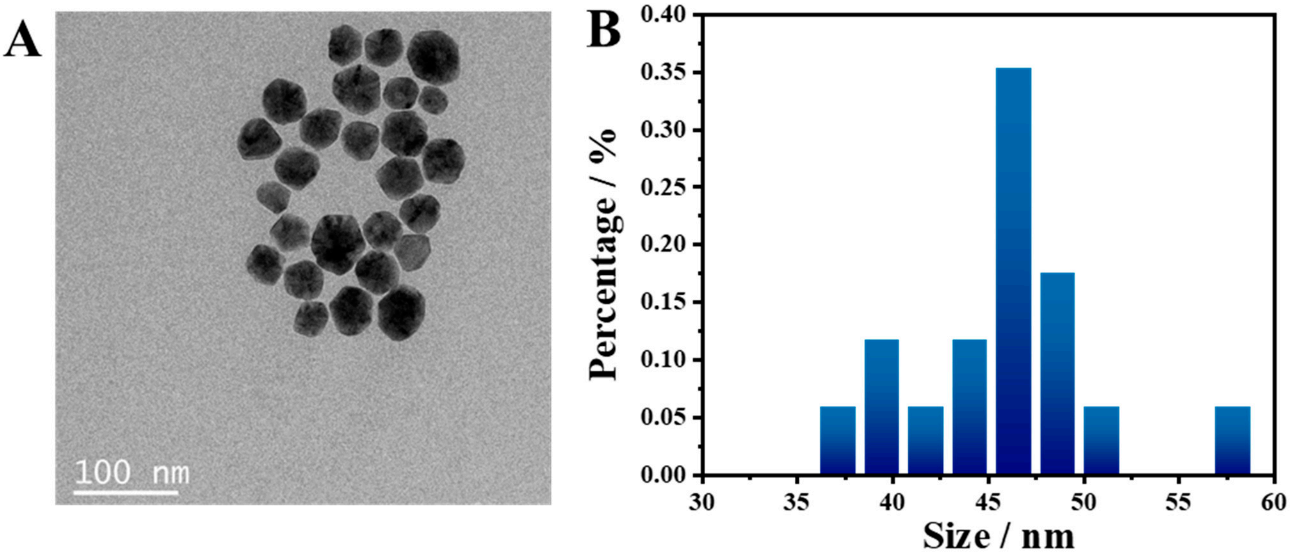

Figure S2 (A) TEM and (B) size distribution of Ag@AuNPs.

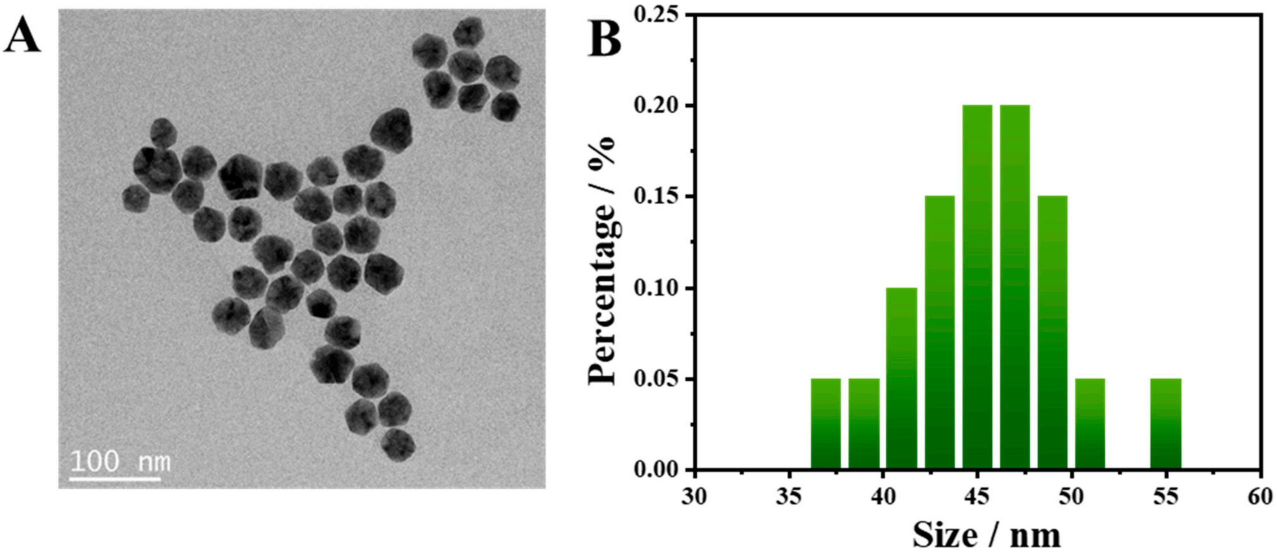

Figure S3 (A) TEM and (B) size distribution of Ag@MBN@AuNPs.

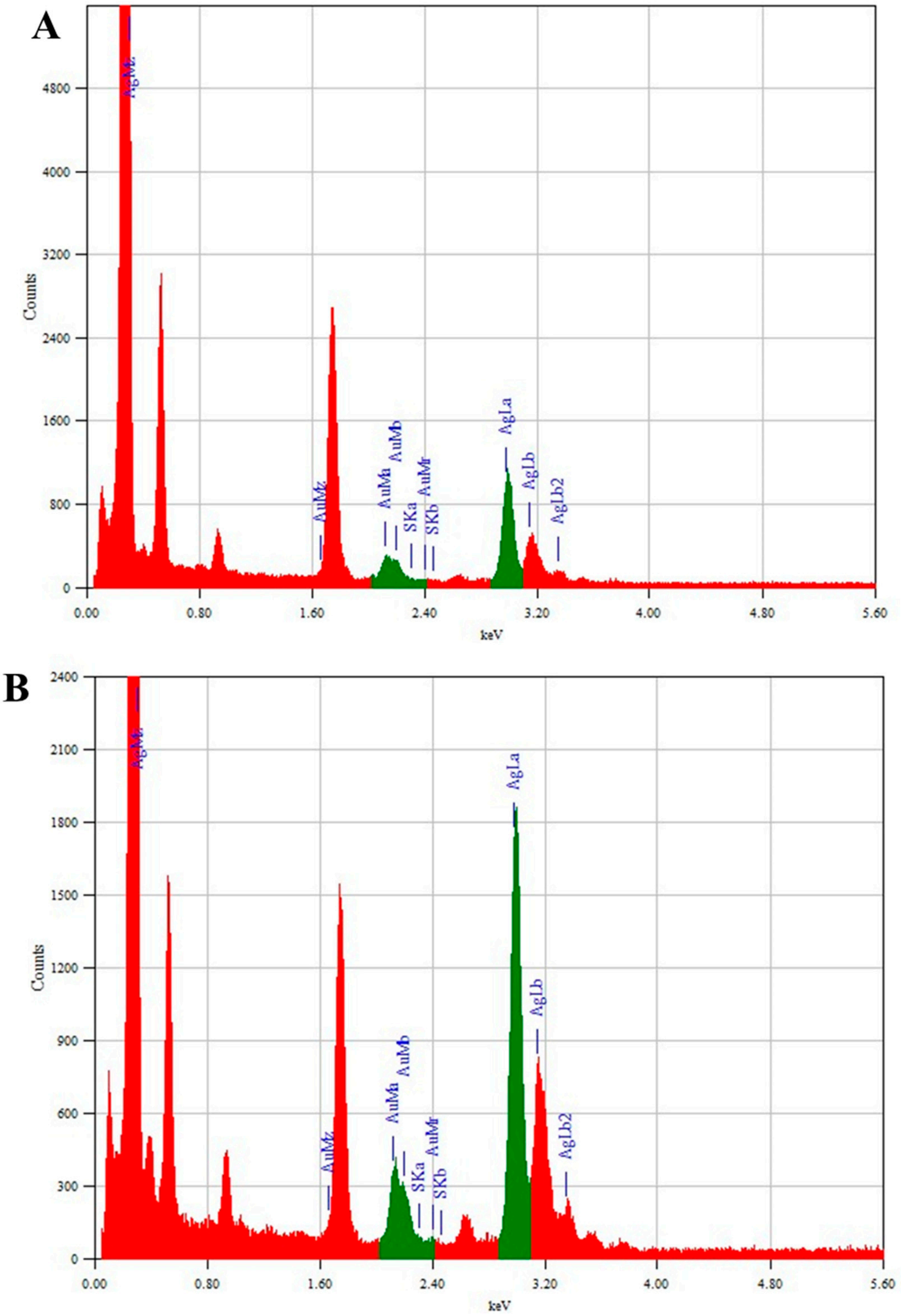

Figure S4 Energy spectrum of (A) Ag@AuNPs and (B) Ag@MBN@AuNPs.

Table S1 Energy spectrum quantification report.

	Ag (Mass%)	Au (Mass%)	S (Mass%)
Ag@AuNPs	87.28	12.72	ND
Ag@MBN@AuNPs	88.39	11.52	0.09

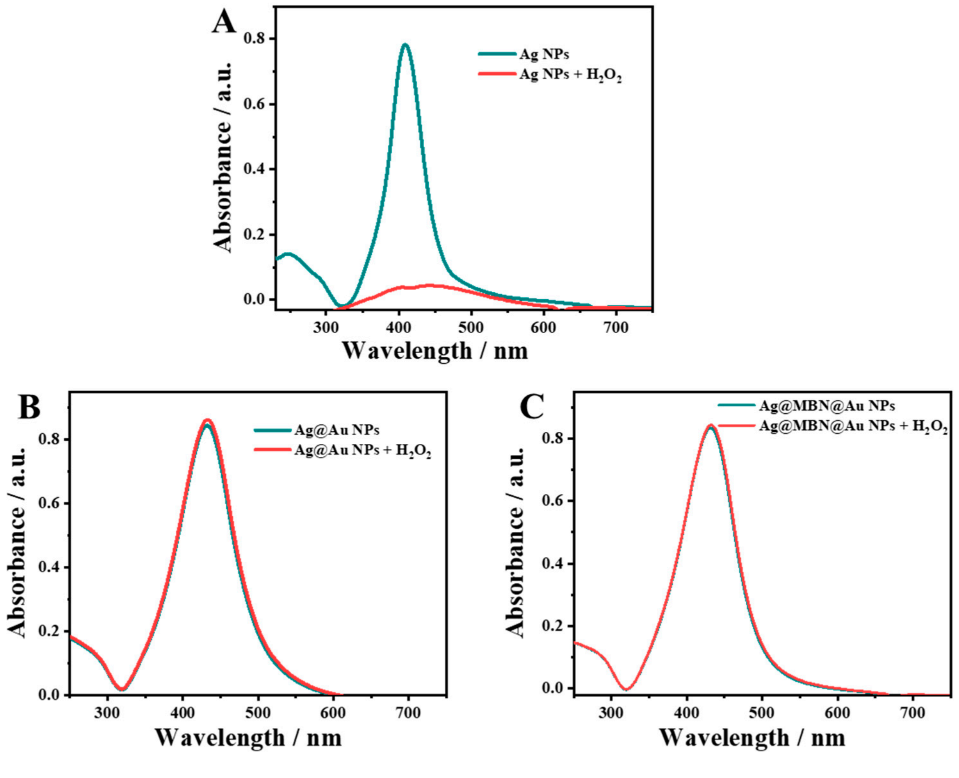

Figure S5. UV−vis spectra of (A) AgNPs, (B) Ag@AuNPs and (C) Ag@MBN@AuNPs before and after treatment with H_2O_2. (1mM).

Table S2 SERS bands assignment of SERS spectra observed in Fig 2 and Fig 3.[1-4]

Raman shift (cm^{-1})	vibrational assignment
993	B–O symmetric stretching
1070	C–H in-plane deformation coupled with C−S stretching vibrations
1582	C=C stretching vibrations
2222	C≡N stretching vibration

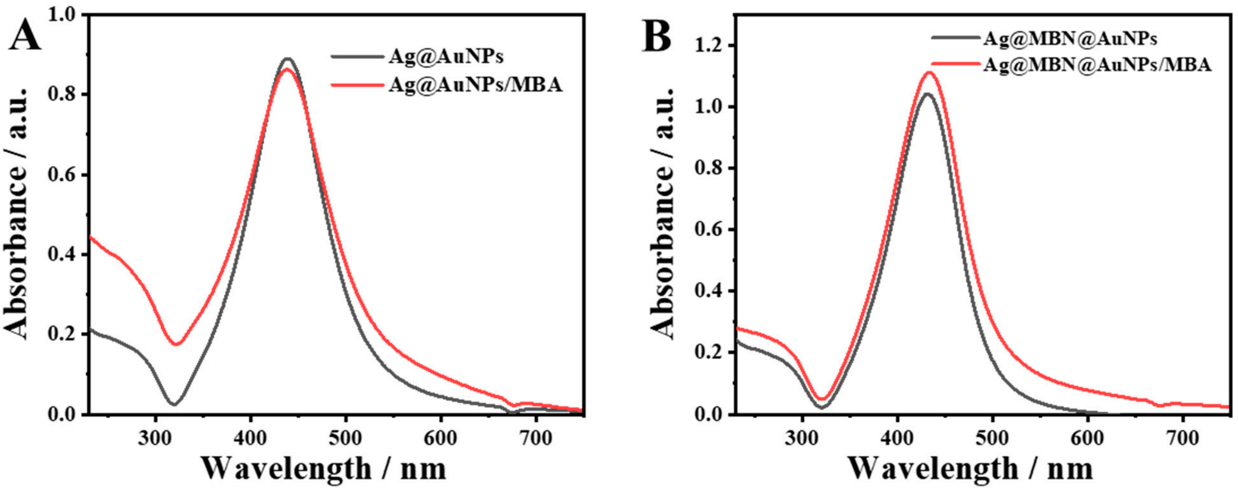

Figure S6. UV−vis spectra of (A) Ag@AuNPs and (B) Ag@MBN@AuNPs before and after modified by MBA

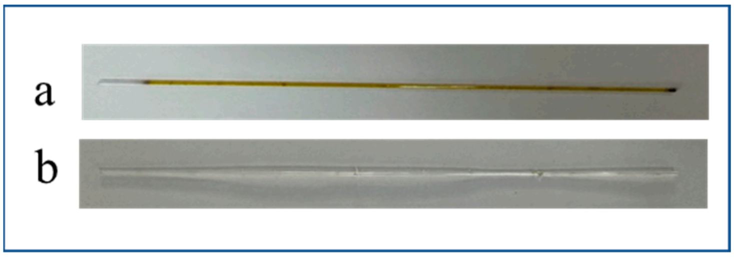

Figure S7. Photographs of aminosilylated capillary treated with Ag@AuNPs (a) and raw capillary treated with Ag@AuNPs (b).

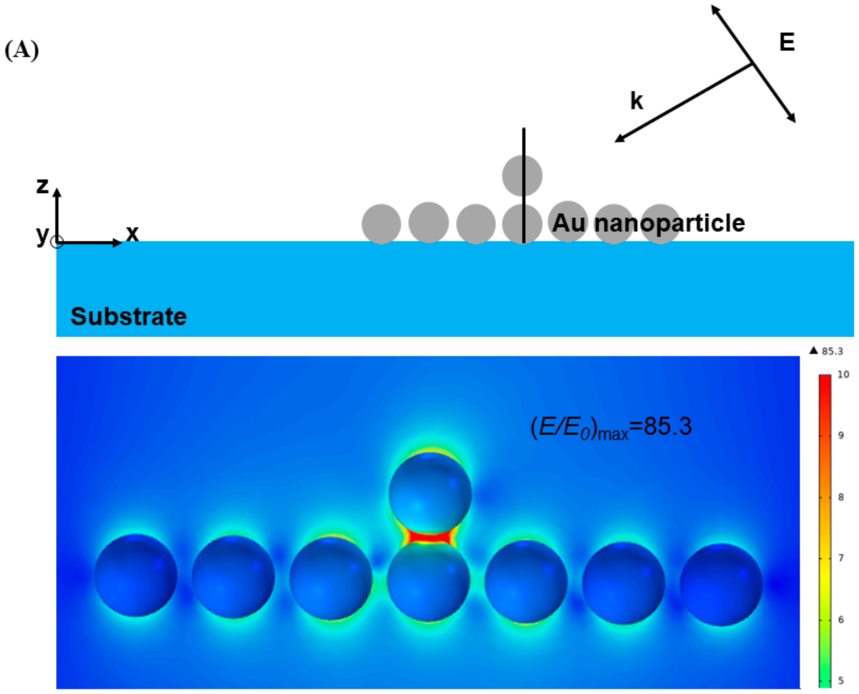

Figure S8 Simulated near-field electromagnetic field distributions of the SERS sensing platform. The distance between the SERS tags and the Ag@AuNPs-layer is about 2 nm calculating based on the molecular structure.

Note: The simulations were performed using the COMSOL software to obtain electric field distributions under 60 deg oblique incidence. Total-field scattered-field source is employed in the simulation. The excitation wavelength was set at 532 nm with the electric field polarized along the x-axis. The complex dielectric constants of the materials were obtained from the database of Johnson-Christy (The Ag@AuNPs were assumed to be Au, and the

size was set to be 46 nm) and Palik (SiO_2, substrate), respectively, perfectly matched layer was used in both three directions and a mesh size of 1.5 nm for the structure region was utilized.

References

1. He X. N.; Wang Y. N.; Wang Y.; Xu Z. R. Accurate quantitative detection of cell surface sialic acids with a background-free SERS probe, *Talanta*, **2020**, *209*, 120579.
2. Muhammad P.; Tu X. Y.; Liu J.; Wang Y. J.; Liu Z. Molecularly imprinted plasmonic substrates for specific and ultrasensitive immunoassay of trace glycoproteins in biological samples, *ACS Appl. Mater. Interfaces*, **2017**, *9*, 12082-12091.
3. Ma S. S.; Li Q.; Yin Y. M.; Yang J.; Liu D. B. Interference-free surface-enhanced Raman scattering tags for single-cell molecular imaging with a high signal-to-background ratio, *small*, **2017**, *13*, 1603340.
4. Peng R. Y.; Si Y. M.; Deng T.; Zheng J.; Li J. S.; Yang R. H.; Tan W. H. A novel SERS nanoprobe for the ratiometric imaging of hydrogen peroxide in living cells, *Chem. Commun*., **2016**, *52*, 8553-8556.
